# Impact of Buffer Layer Process and Na on Shunt Paths of Monolithic Series-connected CIGSSe Thin Film Solar Cells

**DOI:** 10.1038/s41598-019-38945-5

**Published:** 2019-03-06

**Authors:** Chan Bin Mo, Se Jin Park, Soohyun Bae, Mi-hwa Lim, Junggyu Nam, Dongseop Kim, JungYup Yang, Dongchul Suh, Byoung Koun Min, Donghwan Kim, Yoonmook Kang, Young-Su Kim, Hae-seok Lee

**Affiliations:** 1 Department of Materials Science and Engineering, Korea University, 145 Anam-ro, Seongbuk-gu, Seoul, 136-701 Republic of Korea; 2 Gangwon Regional Division, Korea Institute of Industrial Technology, Gangneung-Si, Gangwon-Do 210-340 Republic of Korea; 3 Photovoltaic Development Team, Samsung SDI Co., Ltd., Cheonan-Si, 331-300 Republic of Korea; 4 Department of Physics, Kunsan National University, Gunsan, 54150 Republic of Korea; 5 Department of Chemical Engineering, Hoseo University, Asan, Republic of Korea; 6 KU-KIST Green School, Graduate School of Energy and Environment, Korea University, 145 Anam-ro, Seongbuk-gu, Seoul, 136-701 Republic of Korea

## Abstract

The illuminated current-voltage characteristics of Cu(In,Ga)(S,Se)_2_ (CIGSSe) thin film solar cells fabricated using two different buffer layer processes: chemical bath deposition (CBD) and atomic layer deposition (ALD) were investigated. The CIGSSe solar cell with the ALD buffer showed comparable conversion efficiency to the CIGSSe solar cell with CBD buffer but lower shunt resistance even though it showed lower point shunt defect density as measured in electroluminescence. The shunt paths were investigated in detail by capturing the high-resolution dark lock-in thermography images, resolving the shunt resistance contributions of the scribing patterns (P1, P3), and depth profiling of the constituent elements. It was found that the concentration of Na from the soda-lime glass substrate played a key role in controlling the shunt paths. In the ALD process, Na segregated at the surface of CIGSSe and contributed to the increase in the shunt current through P1 and P3, resulting in a reduction in the fill factor of the CIGSSe solar cells.

## Introduction

The quality and properties of buffer layers in Cu(In,Ga)(S,Se)_2_ (CIGSSe) solar cells are critical from the viewpoint of the diode junction quality, quantum efficiency, series resistance, and shunt resistance^1–6^. Various efforts have been made to develop materials and processes to improve the band alignment, coverage, and uniformities in the composition and thickness of buffer layers. Among the commonly used buffer layer materials, ZnS has been investigated extensively owing to its low cost, non-toxicity, and good transparency at short wavelengths. In addition, ZnS-based buffers show the highest cell efficiencies among CdS-free buffers. ZnS-based buffers are usually fabricated by chemical bath deposition (CBD) method. However, the CBD method has some limitations such as the difficulty in controlling the uniformity and repeatability and the wastage of a large amount of chemicals. In order to overcome these limitations, the atomic layer deposition (ALD) method, which offers conformal coverage, self-limiting surface reactions, and the ease of controlling the buffer layer composition, has been developed. Thus, the ALD method is a potential alternative to the CBD method for the in-line production of buffer layers. However, there are some limitations of this method. Solar cells with ZnS-based buffer layers developed by the ALD method show low fill factors because of their shunt resistance, which depends on the buffer layer composition. However, the shunt paths of such solar cells have not been investigated in detail^3–5^. Such a low shunt resistance can be crucial in monolithic series-connected solar cells because of the inclusion of new shunt paths such as scribing lines, which are not necessarily observed in lab-scale solar cells. The first scribing pattern (P1) is a region where the electrode, Mo, is removed by a series of laser shots. Thus, any imperfections such as micro-bridging or debris of Mo can be primary source of the shunt paths in P1. In addition, Na from the soda-lime glass substrate can diffuse into CIGSSe absorber layer on P1 more easily than that on Mo electrode layer. The Na concentration difference in CIGSSe absorber layer can affect level of the shunt resistance because it causes change in electrical properties of absorber layer including the carrier concentration. The third scribing pattern (P3) is a region where the TCO/Buffer/CIGSSe layers are removed by a needle. Thus, any residues or micro-bridging of TCO can be primary source of the shunt path in P3. In addition, the mechanical scribing using a needle can cause formation of many defects in the buffer layer which prevents shunting between the TCO and the CIGSSe absorber layer.

In this paper, we used the ALD method to deposit a conformal and uniform buffer layer, Zn(S,O) thin film on CIGSSe absorber layer. The characteristics of CIGSSe solar cell with ALD buffer were investigated and compared to CIGSSe solar cell with CBD buffer. In order to explain the difference in fill factor loss mechanism of the CIGSSe solar cells via two different buffer layer processes, the shunt paths were investigated in detail by capturing the high-resolution dark lock-in thermography (DLIT) images, resolving the shunt resistance contributions of the scribing patterns (P1, P3), and depth profiling of the constituent elements.

## Results

### Microstructures of the buffer layers

**Figure 1:**
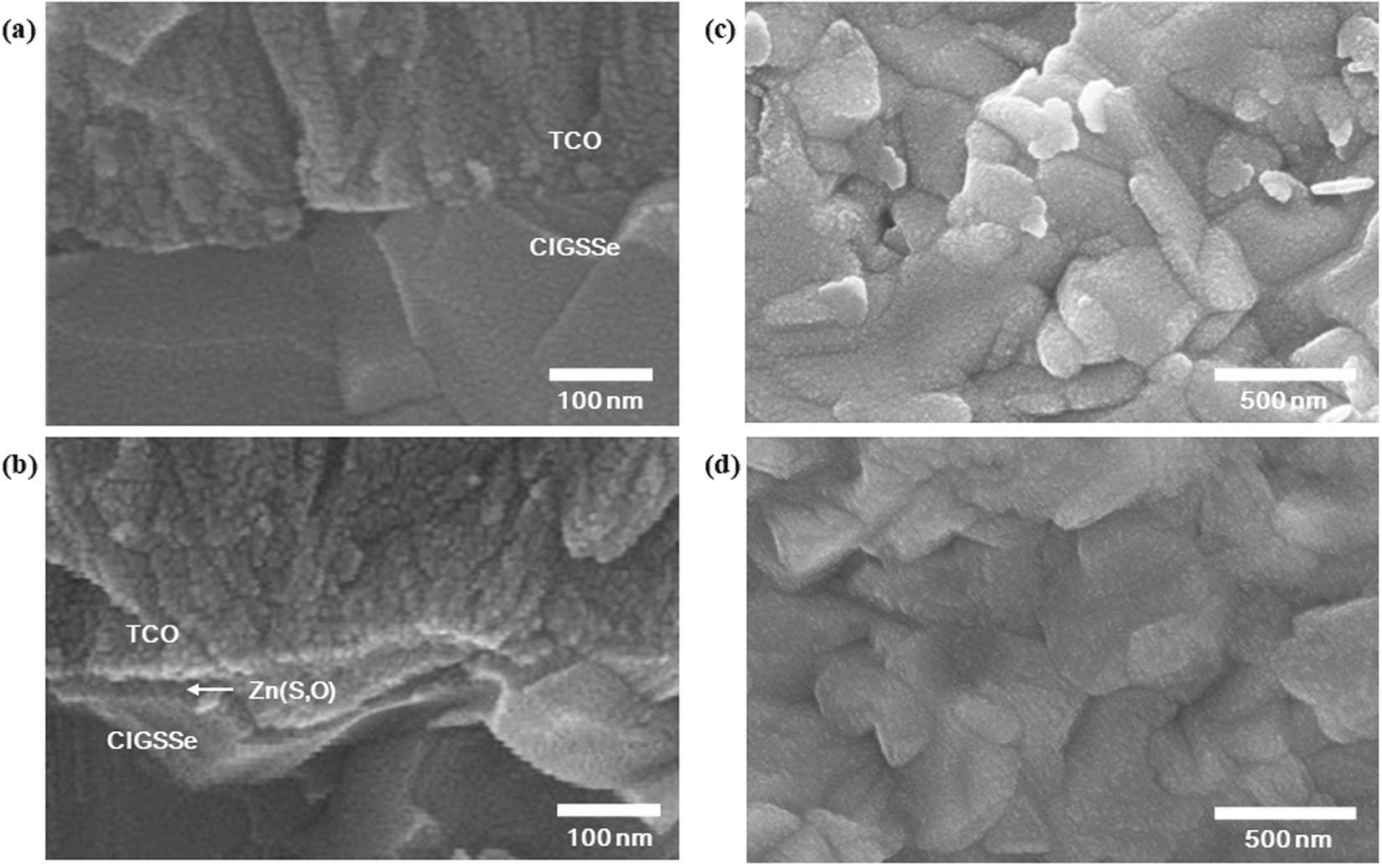
SEM images of the CIGSSe solar cells with two different buffer layers, cross-sectional view images of (**a**) the CBD buffer and (**b**) the ALD buffer, top view images of (**c**) the CBD buffer and (**d**) the ALD buffer on CIGSSe.

Figure 1 shows the cross-sectional scanning electron microscopy (SEM) image of the buffer layers after cleaving the completed CIGSSe solar cells with the transparent conductive oxide (TCO) layer and the top view of the buffer layers on the solar cells immediately after each buffer process. The thickness of the CBD buffer could not be accurately measured from the SEM image, while the thickness of the ALD buffer, as indicated by the arrow in Fig. 1(c), was continuous throughout the sample and was almost equal to the designed thickness (30 nm). From the top view images of the buffer layers, it can be observed that both of the CBD and ALD buffer layer showed a good coverage throughout the CIGSSe absorber layer. The sulfur to oxygen (S/(S + O)) atomic ratio and thickness of the ALD buffer layers was controlled by adjusting the gas pulsing ratio and cycle numbers, as reported previously by other researchers^2–5^. The S/(S + O) atomic ratio was about 0.27 and the thickness was 30 nm (Supplementary Fig. S1) when H_2_S/(H_2_S + H_2_O) gas pulse ratio was 1/4. The calibrated X-ray fluorescence (XRF) measurements revealed that the thickness of the CBD buffer was about 5 nm. The 5G-scale (900 mm × 1600 mm) thickness uniformity of ALD buffer layers (20%) were slightly better than CBD layer (25%) (Supplementary Figs S1 and S2).

### Illuminated I–V characteristics

**Figure 2:**
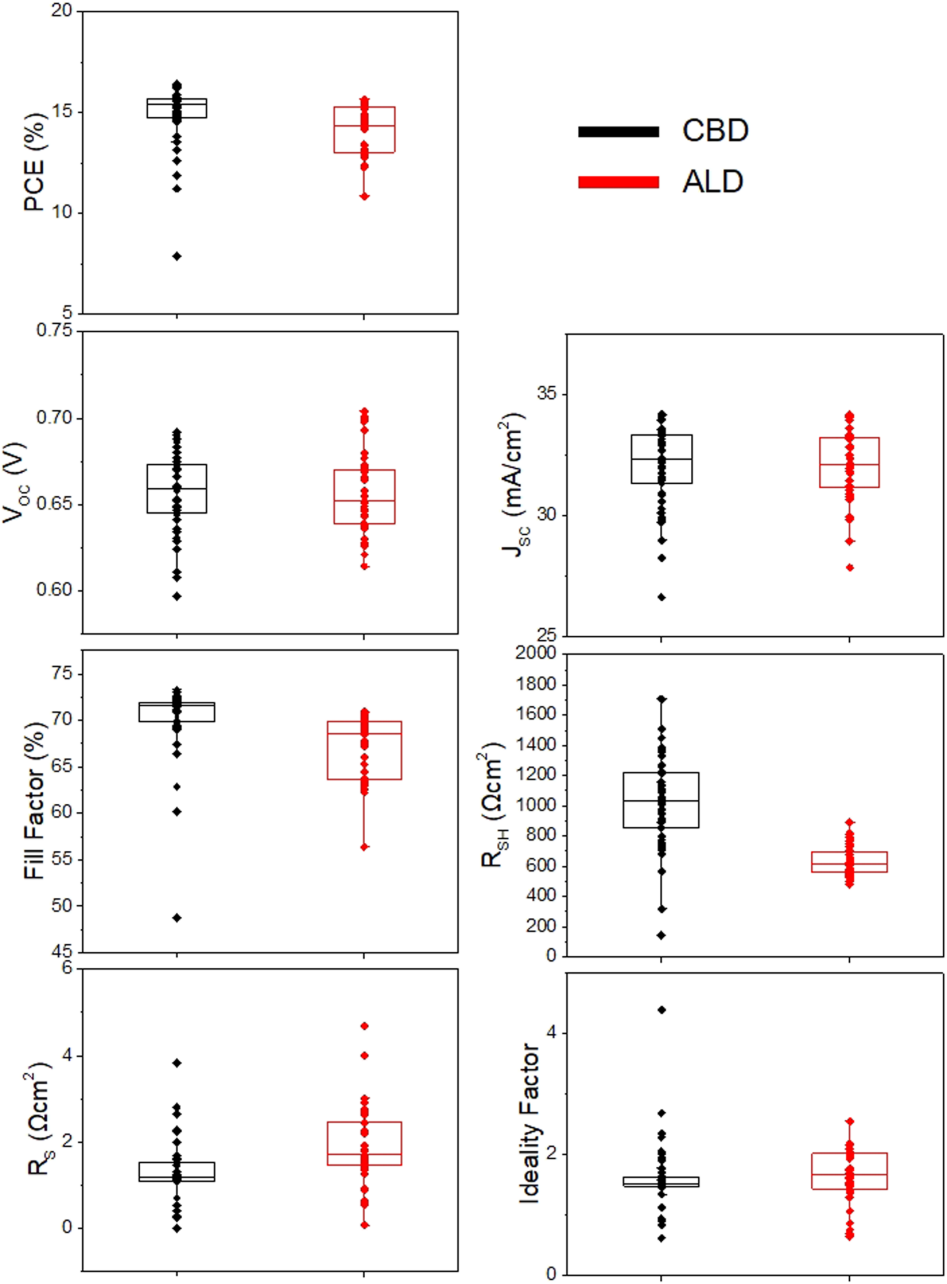
Illuminated I–V parameters of the CIGSSe solar cells with CBD and ALD buffers fabricated in three distinct process batches.

At 5G scales, the uniformity issues of thin films always exist not only within the glass but also between the process batches. Thus, the CIGSSe solar cells were fabricated three times using the same process but in different batches in order to compare the characteristics of the CIGSSe solar cells with CBD and ALD buffer layers by means of statistical analysis. The illuminated I–V parameters of the CIGSSe solar cells (with the dimensions of 300 mm × 320 mm evenly cleaved from the corresponding 5G-size solar cells) were measured and calculated by using Sunshade method^7^. Figure 2 shows the box plots of the photo conversion efficiencies, open circuit voltages, short circuit currents, fill factors, shunt resistances, series resistances, and diode ideality factors for the CIGSSe solar cells with the CBD and ALD buffer layers.

It is noteworthy that the CIGSSe solar cell with the ALD buffer showed lower shunt resistance and higher series resistance and diode ideality factor than the CIGSSe solar cell with the CBD buffer. The open circuit voltages and short circuit currents of the two solar cells were similar. The CIGSSe solar cell with the ALD buffer showed higher series resistance because of its higher buffer thickness. This was confirmed by varying the thickness of the ALD buffer layer from 5 to 40 nm (Supplementary Fig. S3). Lavrenko *et al*. reported that the series resistance of sputtered Zn(O,S) buffer layers increases with an increase in the sulfur content to about 40%^6^. In this study, the sulfur content of the ALD buffer was about 27%, and hence its effect on the series resistance was not significant. However, the lower shunt resistance of the solar cell with the ALD buffer in spite of the higher thickness and better uniformity of the ALD layer than that of the CBD layer as shown in Fig. 1 and XRF measurement is difficult to explain. Kobayashi *et al*. reported values of shunt resistance (below 1,000 Ωcm^2^) at the H_2_S/(H_2_S + H_2_O) ratio of 0.06 and 0.11^3^. They mentioned that the recombination at the absorber/buffer interface can cause change in the shunt resistance but did not investigate the shunt paths and mechanisms in detail. Such low shunt resistances can affect the fill factor loss of solar cells, which is calculated using the equations reported by Green *et al*. The fill factor loss by the shunt resistance increases with a decrease in the shunt resistance and the absolute value becomes larger than 1% when the shunt resistance is decreased to less than 1,000 Ωcm^2^, which is critical for obtaining high-efficiency solar cells^8^. The deviation becomes much larger than 2% when the shunt resistance becomes less than 500 Ωcm^2^ (Supplementary Table S1). In addition, such a low shunt resistance can cause overestimation of the diode ideality factor when they are measured by Sunshade method. This is because under shade conditions, the low shunt resistance of solar cells decreases their open circuit voltage, as reported by Park *et al*.^7^. Thus, high diode ideality factor of CIGSSe solar cell with ALD buffer is attributed not to junction recombination but to the measurement error caused by low shunt resistance.

Normally, it is believed that CIGS solar cells with thick buffer layers show high shunt resistances because of the formation of a large number of Ohmic shunt paths and a weak diode by the locally thin and blank buffer layer. This simple relationship between the thickness of a buffer layer and its shunt resistance is valid only when the contributions from the other shunt paths such as pinholes in absorber layer and scribing defects are negligible. However, in the case of monolithic series-connected solar cells, the imperfections in the scribing patterns such as P1 and P3 can cause low shunt resistance, resulting in a significant decrease in the fill factor.

### Analysis of shunt paths by imaging technologies

**Figure 3:**
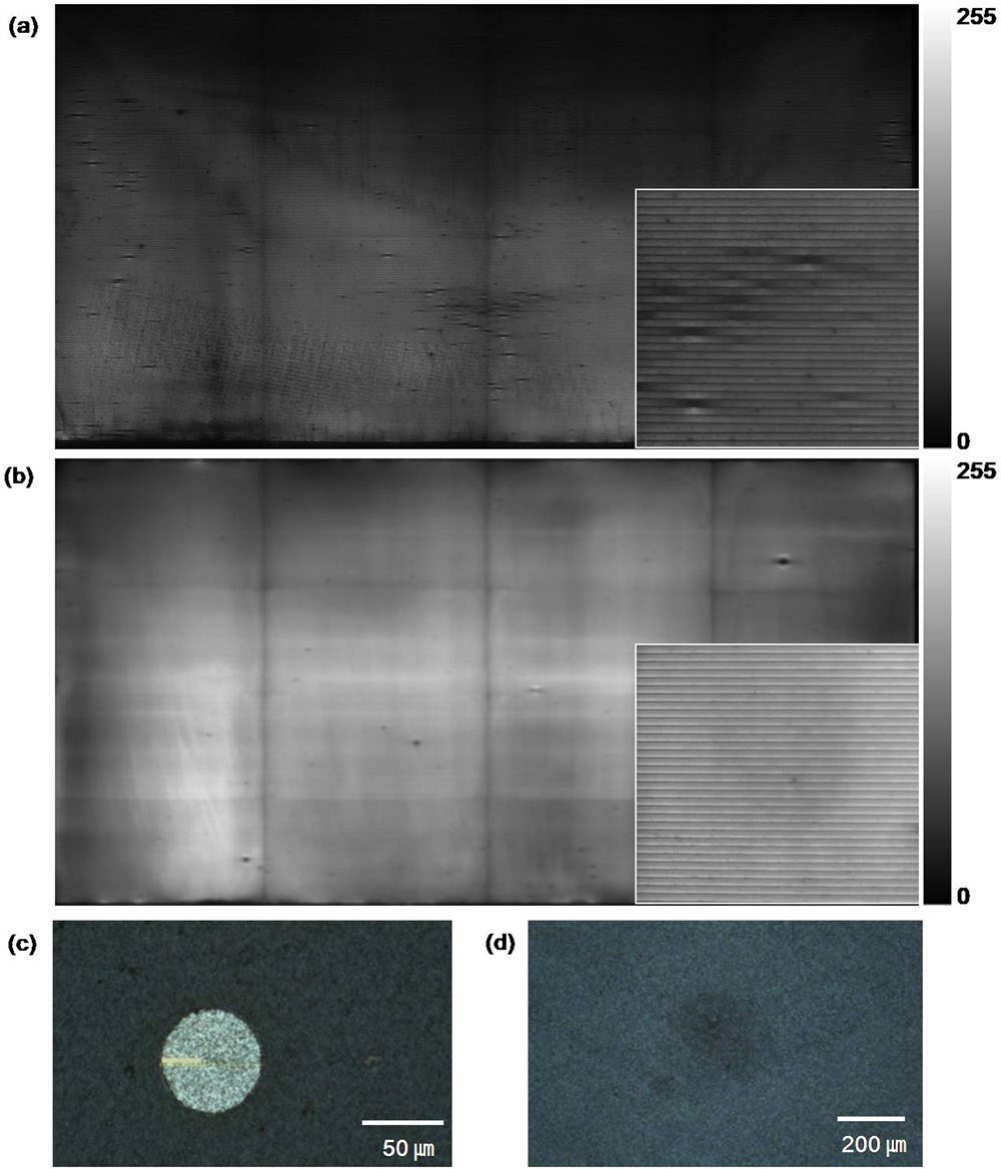
EL image of CIGSSe solar cells with the (**a**) CBD buffer, (**b**) ALD buffer and (**c**), (**d**) microstructures of the point defects in (**a**).

**Figure 4:**
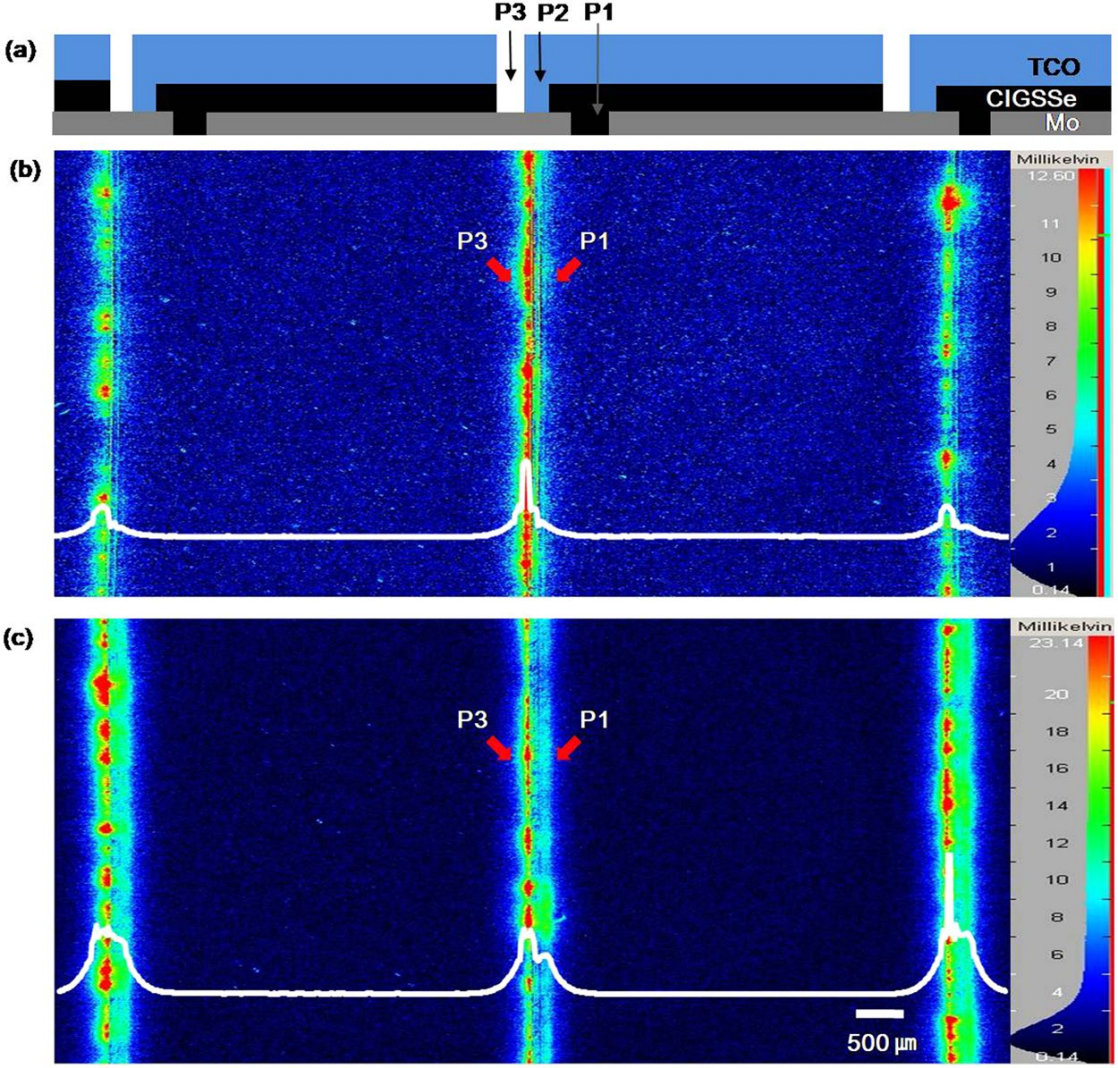
CIGSSe solar cell coupon (**a**) and DLIT images at reverse bias for the CIGSSe solar cell with (**b**) the CBD buffer and (**c**) the ALD buffer. The bottom white lines are the horizontal line profiles of temperature throughout the samples.

Figure 3(a,b) show the electroluminescence (EL) images of the CIGSSe solar cells with the two different buffer layers. The CIGSSe solar cell with the CBD buffer layer (Fig. 4(a)**)** showed many point defects (dark spots in the inset). These point defects were mainly caused by the pinholes in the CIGSSe layer and the nodule-type defects (Fig. 4(c,d)**)**, which have also been reported by other researchers^9,10^. Some of the defects originated from P1 and P3 could be distinguished as the crosstalk with the neighbouring cells in the EL image, as reported by Misic *et al*.^11,12^. The broad dark area in the EL image can be attributed to the inhomogeneities of the CIGSSe layer such as low band gap and lifetime or to the high series resistance. The EL of the solar cells was measured using a Si-based charge-coupled device as it is described in Method section. Thus, if the band gap of CIGSSe is lower than the Si the radiation photon energy is not enough to excite the Si CCD. The radiative recombination rate in a semiconductor is proportional to the recombination constant and the excess carrier density. However, other recombination mechanisms such as Shockley-Read-Hall (SRH) and Auger recombinations show much higher recombination rate than radiative recombination, which result in low carrier lifetime. Thus, the EL intensity depends on the carrier lifetime when SRH recombination dominates the radiative recombination due to high density of defects in CIGSSe absorber layer. However, the detailed analysis of this dark area is beyond the scope of this study.

On the other hand, in the case of the CIGSSe solar cell with the ALD buffer, there were only a few defects, which were very small as compared to those of the CBD buffer (Fig. 3(b)**)**. It indicates that the conformal 30 nm-thick ALD buffer layer prevented the formation of point defects. Nevertheless, the shunt resistance of the CIGSSe solar cell with the ALD buffer layer was much lower than that of the CIGSSe solar cell with the CBD buffer layer.

In order to find the cause of the low shunt resistance of the CIGSSe solar cell with the ALD buffer layer, we captured the high-resolution dark lock-in thermography (DLIT) images of both the CIGSSe solar cells (Fig. 4**)**. Figure 4(a) shows the location of P1 and P3 in the CIGSSe solar cells. The temperature change and joule heating caused by the shunting current through the active cell and scribing patterns (P1, P3) were observed at reverse bias. A reverse bias of −90 V was used for all the samples with 65 cells in a series connection. The peak temperature of the CIGSSe solar cell with the CBD buffer was 12 mK, while that of the CIGSSe solar cell with the ALD buffer was 23 mK. This difference in the peak temperatures of the CIGSSe solar cells can be attributed to the difference in their shunt resistances. At a given bias, a low shunt resistance gives rise to a high current, thus enhancing the joule heating, which is proportional to the square of the current. In the case of the CIGSSe solar cell with the ALD buffer layer, the thermal heating through both P1 and P3 could be clearly seen. On the other hand, in the case of the CIGSSe solar cell with the CBD buffer layer, the thermal heating through P1 was not clear (Fig. 4(b,c)**)**. Misic *et al*. reported experimental and simulated DLIT images with various shunting paths through P3 and P1, and bright spots and heat centres could be observed when the shunting paths were formed by bridging between the Mo or TCO layers^11,12^. However, the DLIT images obtained in this study did not match with any of the simulated cases, indicating that the shunt paths in this study were not formed by a direct Ohmic contact between the conductors (Mo, TCO).

**Figure 5:**
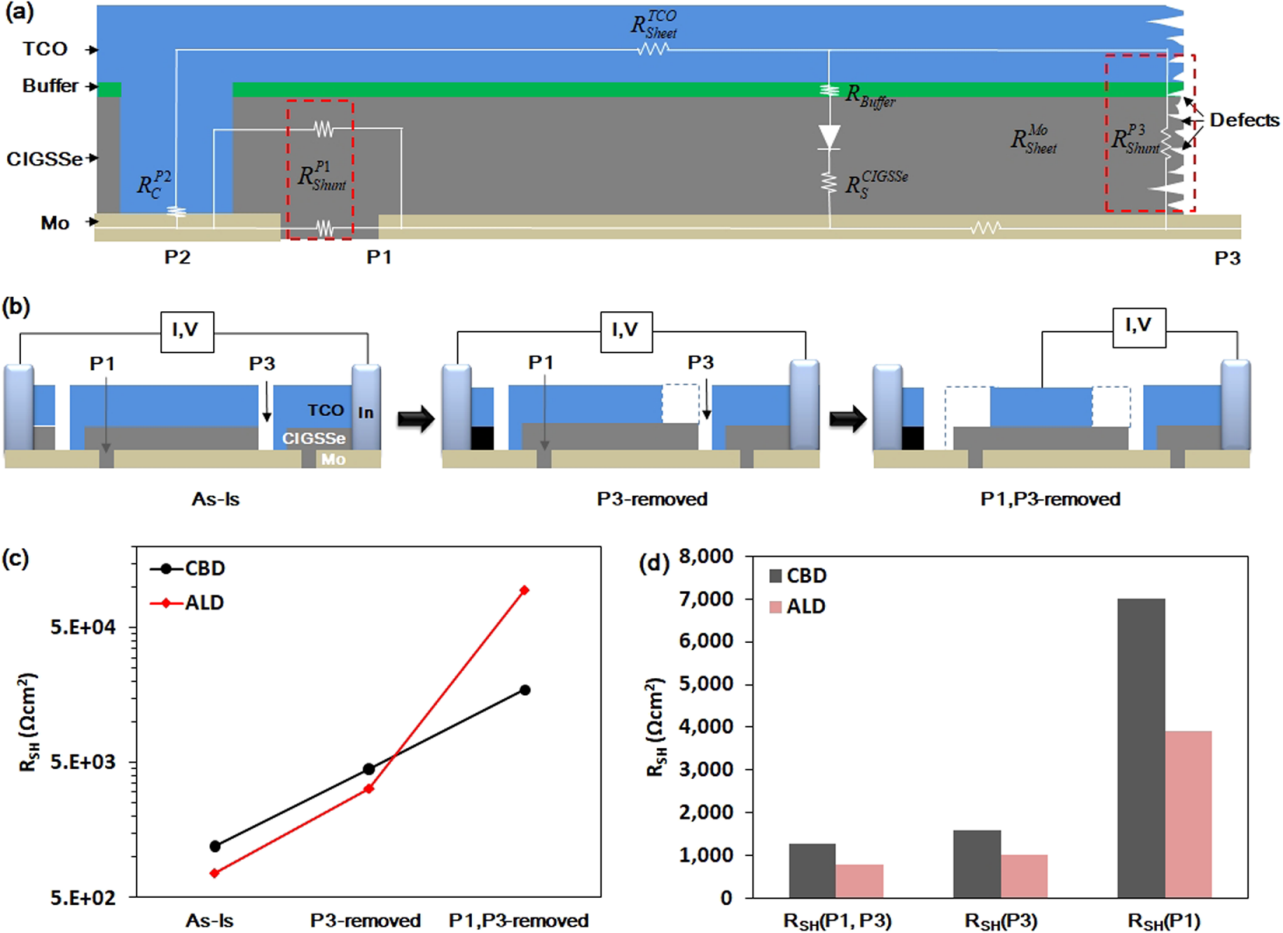
(**a**) Schematic for the shunt paths, (**b**) measurement of the shunt resistances by sequentially etching out TCO over P3 and P1, (**c**) the measured shunt resistance, and (**d**) calculated shunt resistance contributions of P1 and P3 (R_SH_(P1, P3), by only P3(R_SH_(P3) and by only P1(R_SH_(P1)).

When shunting was done through P1, the only remaining shunt path was CIGSSe itself if the Mo layer was well separated. The difference in the conductivity of CIGSSe at various compositions can result in different degrees of shunting at P1. The current paths through P1: 1) Mo(left cell)-CIGSSe(bottom)-Mo(right cell), 2) Mo-CIGSSe(top)-TCO are shown in Fig. 5(a). Both the paths did not have any buffer layer or electronic barrier to prevent shunting.

### Quantitative measurement of shunt resistance contributions

It is not easy to quantitatively determine the shunt resistance contributions from P1 and P3 directly from the DLIT images. Thus, a new approach was needed to break down the shunt paths and their contributions. Allsop *et al*. reported that the fill factor of solar cells changes dramatically during the damp heat tests and recovers after rescribing by the removal of the damaged cell portions around the scribe line^13^. However, new scribing lines also can be new shunting paths. Hence, we used another approach to remove the current paths through the scribing lines.

In the case of shunting at P3, the current flowed through the TCO layer, and thus, the shunt path could be effectively eliminated by removing the TCO layer on P3. In the case of shunting at P1, the current flowed through TCO and the series-connected Mo. Hence, the shunt path could be effectively eliminated by removing the TCO layer on P1 and probing the TCO layer at the centre of the solar cell, as shown in Fig. 5(b). The TCO layer (0.5 mm from the scribing line edge) on each pattern was etched out by HCl (5%, at room temperature) while the other regions were masked using Polyimide tape. The shunt resistance was measured by fitting the dark I–V curve using a one-diode model and the newly defined cell area. Using this approach, the contribution of each shunting path could be defined quantitatively (detailed calculations are described in Supplementary Table S2). The removed area of TCO includes active area of solar cell beside the scribing line which can results in errors in shunt resistance measurement. However, the width of thermal heating at P1 and P3 was less than 0.5 mm and the magnitude of the temperature is one order larger than that of the active area, as shown in the DLIT images. Thus, it can be assumed that the shunting current through the removed active area near the scribing line was almost negligible.

Figure 5(c,d) show the measured and calculated shunt resistance contributions of P1 and P3, respectively. The shunt resistances of the as-prepared CIGSSe solar cell coupons (10 mm in length) with the CBD and ALD buffer layers were 1,194 Ωcm^2^ and 759 Ωcm^2^, respectively. When the TCO layer on P3 was etched out (P3-removed Case), the shunt resistances of the CIGSSe solar cells with the CBD and ALD buffer layers were 4,476 Ωcm^2^ and 3,224 Ωcm^2^, respectively. The shunt resistance contribution of P3 was calculated to be 1,569 Ωcm^2^ and 1,001 Ωcm^2^ for the CIGSSe solar cells with the CBD and ALD buffer layers, respectively. When the TCO layers on both P3 and P1 were etched out (P1 and P3-removed Case), the shunt resistances for the CIGSSe solar cells with the CBD and ALD buffer layers were 17,672 Ωcm^2^ and 95,646 Ωcm^2^, respectively. It is surprising that the CIGSSe solar cell with the ALD buffer layer showed a very high shunt resistance (almost five times higher than that of the CIGSSe solar cell with the CBD buffer layer). It means that the ALD buffer prevents any tunnelling and recombination effectively. The contribution from P1 was calculated be 6,984 Ωcm^2^ for the CIGSSe solar cell with the CBD buffer layer and 3,898 Ωcm^2^ for the CIGSSe solar cell with the ALD buffer layer. The contribution from both P1 and P3 was calculated to be 1,274 Ωcm^2^ for the CIGSSe solar cell with the CBD buffer layer and 766 Ωcm^2^ for the CIGSSe solar cell with the ALD buffer layer. On the basis of these results, it can be said that the shunt resistance from the scribing patterns was more severe in the case of the ALD buffer than that in the case of the CBD buffer. Any compositional change in the solar cell layers can affect the parasitic current paths. Hence, depth profiling was carried out by secondary ion mass spectroscopy (SIMS).

### Origin of low shunt resistance in the CIGSSe solar cell with the ALD buffer

**Figure 6:**
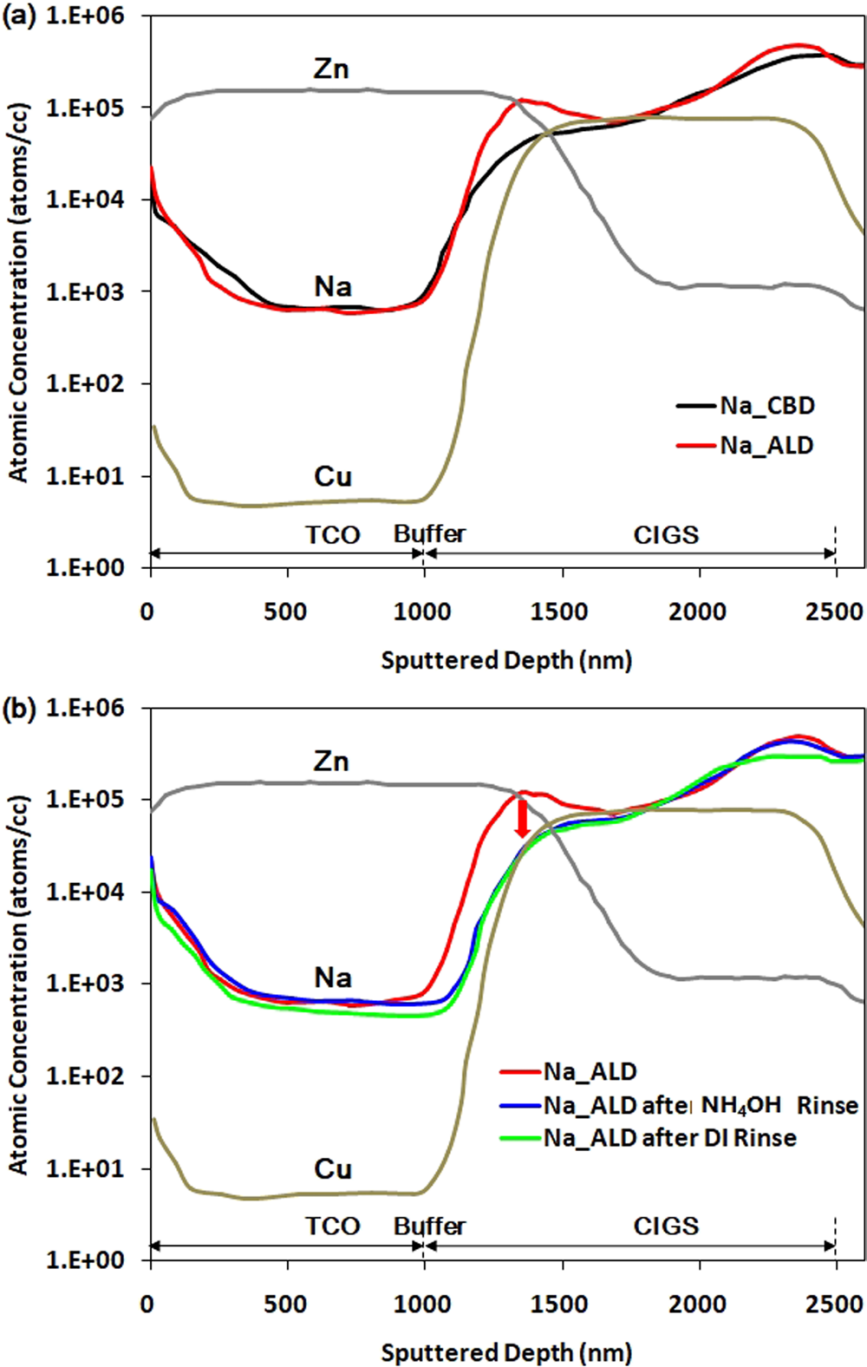
Depth profile for (**a**) the CIGSSe solar cells with CBD and ALD buffers obtained by SIMS, (**b**) ALD buffer after rinsing in DI water and NH_4_OH baths.

Figure 6 shows the depth profile of CIGSSe solar cell focused on Na which is originated via diffusion from the soda-lime glass substrate and affects electrical properties of CIGGSe absorber layer as mentioned in the introduction part. CIGSSe absorber layers for the CBD and ALD buffers were prepared in the same batch using the same glass, Mo, precursor, and selenization/sulfurization processes. Hence, the compositions of Cu, In, Ga, Se, and S were the same for both the layers. In Fig. 6(a) it can be seen clearly that at the concentration of Na at the surface of the CIGSSe layer was much higher than that in the bulk region. This is similar with the results reported previously^14–17^. Furthermore, the surface concentration of Na was higher in the ALD buffer than that in the CBD buffer. The thickness of the Na-rich CIGSSe layer (compared to CBD buffer) was almost 500 nm, which is about 1/3 of the total thickness of the CIGSSe layer. It was expected that the electrical properties of the CIGSSe layer can be affected significantly if the Na-rich surface layer has different electrical properties compared to normal layer. Theelen *et al*. reported that the degradation of Na-rich CIGSe_2_ solar cells affects their shunt resistance by the accumulation of Na^+^ at the depletion region, which results in the formation of shunt paths and a subsequent decrease in the shunt resistance from 2,505 Ω to 9 Ω^17^. Na-poor samples on the other hand, showed a small change from 1,787 Ω to 135 Ω. Rockett reported that in polycrystalline CIGS layers, Na tends to accumulate at the grain boundaries and reduces the compensating defects and usually enhances the carrier concentrations, resulting in a decrease in the resistivity of the grain boundaries^14^. Based on the SIMS profiles of the CIGSSe layers and the previous reports, it can be assumed that the shunt resistance through the P1 region was reduced by the low resistivity of the CIGSSe layer caused by reduction of the internal field in the depletion region or by an increase in the conductivity of the grain boundaries by Na.

Witte *et al*. reported that annealing at elevated temperatures (200 °C or 300 °C) induces the migration of Na to the absorber/Zn(O,S) buffer interface^16^. However, in this study, the temperature required for the ALD process was only 110–120 °C. Theelen *et al*. reported that the presence of water enables the release of Na^+^ ions through the hydrolysis of the bonds between CIGS and Na^17^. Rockett proposed that Na can be effectively removed from the surface of CIGS polycrystals by a water rinse^14^. However, there are no specific reports on this phenomenon. Thus, in order to investigate the origin of the difference in the Na concentration at the surface of CIGSSe with the two buffer layers, we introduced an additional step for the fabrication of the CIGSSe solar cell with the ALD layer (between the CIGSSe preparation and ALD). The CIGSSe layer was rinsed in deionized(DI) water (60 °C, 8 minutes) and NH_4_OH(1 M, 60 °C, 8 minutes) baths, which were also used in the CBD process. Figure 6(b) shows the depth profile of Na for the CIGSSe solar cells with the ALD buffer after rinsing in DI water and NH_4_OH baths. The figure shows that in both the baths, reduction in the Na concentrations at the CIGSSe surface was similar to that observed in the case of CBD.

**Figure 7:**
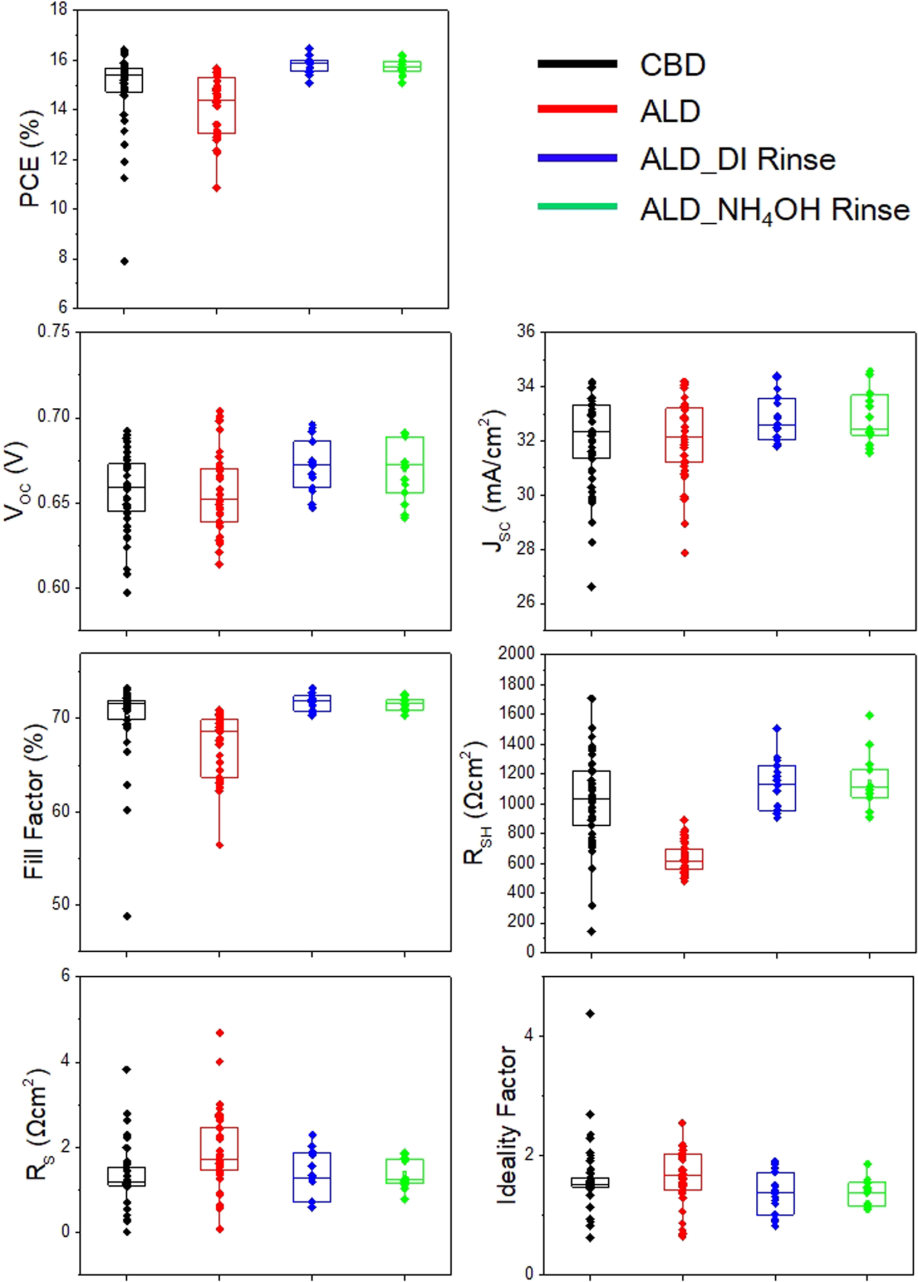
Illuminated I–V parameters of the CIGSSe solar cells with CBD and ALD buffers and ALD buffer after cleaning in DI water and NH_4_OH baths.

As a result, the shunt resistance median of CIGSSe with the ALD buffer increased to more than 1,000 Ωcm^2^. This value is similar to the shunt resistance median of the solar cell with the CBD buffer (Fig. 7**)**. In addition to the shunt resistance, the other parameters including the diode ideality factor, fill factor, open circuit voltage, short circuit current, and conversion efficiency were improved significantly.

## Discussion

The improved performance of the CIGSSe solar cell can be mainly attributed to the removal of shunt paths. Theelen *et al*. reported performance degradation due to the effect of Na^+^ ions on the internal electrical field of the depletion region and the shunt spots on the surface of TCO^17^. However, in this study, it was hard to find shunt paths which were observed as the formation of spots as they reported. Timmo *et al*. reported that NH_4_OH etches Cu and the detrimental secondary phases, which can affect the quality of heterojunctions^18^. However, in this study, rinsing with DI water which cannot etch Cu showed the same effect with rinsing at NH_4_OH. Thus, the etching of Cu and other secondary phases was likely not the main reason for the improved performance of the CIGSSe solar cell with the ALD buffer. On the basis of the results obtained from the quantitative measurement of the shunt resistances at each scribing pattern and Na profiles under different rinsing conditions, it can be stated that the dominant shunt paths of the CIGSSe solar cell with the ALD buffer layer were P1 and P3 scribing patterns because of the high concentration of Na at the CIGSSe surface. The other shunt paths such as the Ohmic point defects and weak diodes were effectively blocked by the 30 nm-thick conformal ALD buffer layer as shown in Fig. 3. Figure 5(a) shows the schematic of the shunt paths through P1. The CIGSSe surface region at P1 was the Mo-to-TCO current path. There was no buffer layer between the edge of the Na-rich CIGSSe layer and TCO because TCO was formed after P2 mechanical scribing, which removed the CIGSSe/buffer multilayer. Furthermore, the thickness of the Na-rich CIGSSe layer was almost 1/3 of the total CIGSSe thickness, as shown in the depth profiles. Thus, the effect of this layer was significant and the resistance of the CIGSSe layer at P1 region, ${{\rm{R}}}_{{\rm{shunt}}}^{{\rm{P}}1}$ could be almost halved (from 6,984 Ωcm^2^ to 3,898 Ωcm^2^), as shown in Fig. 5. Many defects existed in the P3 region because of the mechanical scribing of the CIGSSe/buffer/TCO multilayer, which resulted in the formation of cracks and residues and the intermixing of the layers. This resulted in the formation of parasitic currents paths such as weak diodes or tunneling^9,19^.

It is well known that the quality of ALD buffers depends on the surface conditions of the absorber. Hariskos *et al*. reported that the absorber storage history is crucial for device performance^1^. They reported that vacuum storage is the best method to obtain the optimal surface conditions for absorbers. On the other hand, Hong *et al*. etched the absorber layer, Cu_2_ZnSn(S,Se)_4_ by KCN just before the samples were transferred to the ALD chamber^5^. None of them reported the effects of the storage history and pre-treatment processes of absorbers (prior to the buffer layer deposition) on their Na profile. In this study, it was confirmed that Na concentration at the CIGSSe absorber surface is very sensitive to chemicals and it can affect electrical properties of CIGSSe solar cells. The effect of Na varies according to the concentration of Na in the absorber layer depending on the deposition processes. Thus, both of ALD buffer quality and Na profile should be considered for the best performance of the CIGSSe solar cell with ALD buffer.

In summary, we fabricated CIGSSe solar cells with buffer layers via two different processes: CBD and ALD. The CIGSSe solar cell with the ALD buffer showed a significantly lower shunt resistance, and hence a lower fill factor than the solar cell with the CBD buffer. The EL images showed that the CIGSSe solar cell with the ALD buffer had lesser number of point defects than the solar cell with the CBD buffer. In order to investigate the origin of shunt paths, high-resolution DLIT images of the solar cells were captured focusing on the scribing patterns (P1, P3), and the shunt paths were resolved to calculate for the first time, the quantitative contribution of each pattern. It was found that most of the shunt resistance originated from the scribing patterns and their contributions were more dominant in the CIGSSe solar cell with the ALD buffer. Further investigations on the depth profiles of the CIGSSe solar cells revealed that a relatively large amount of Na was segregated at the CIGSSe surface. The surface Na concentration decreased and the shunt resistance increased when the absorber layer was rinsed in DI water or NH_4_OH prior to the ALD process. On the basis of these results, it can be stated that presence of excess Na in the surface layer of the absorber can increase the conductivity of the CIGSSe layer, resulting in an enhanced shunt current through both P1 and P3 although ALD buffers are conformal and effective in preventing the formation of point shunt paths. Therefore, it is recommended to rinse out excess Na, especially in CIGSSe solar cells with ALD buffers and monolithic series-connected solar cells where the parasitic current through the scribing patterns can reduce the shunt resistance and fill factor significantly.

## Methods

### Fabrication of CIGSSe solar cells

The absorber layer, CIGSSe, was prepared as follows. First, the back contact Mo layer was deposited on a 5G-size (1600 mm × 900 mm) soda-lime glass substrate by DC magnetron sputtering. In order to obtain a monolithically integrated solar module, we scribed the Mo layer with a high-power laser for the first pattern (P1). A stack of Cu/Ga/In thin films was deposited by DC magnetron sputtering and was placed in a reaction furnace for the subsequent selenization in a H_2_Se (99.99%) atmosphere and sulfurization in a H_2_S (99.99%) atmosphere with N_2_.

The buffer layers were grown on the CIGSSe layer by two different processes: CBD and ALD. The Zn(S, O,OH)-based buffer layer was deposited using chemicals such as ZnSO_4_, NH_4_OH, and Thiourea dissolved in DI water. The ALD buffer was deposited by pulsing diethyl zinc, H_2_S, H_2_O, N_2_ purge gases at 110–120 °C. The pulse ratio was controlled to maintain a S:O ratio of 1:4.

After the buffer processes, scribing for the second pattern (P2) was carried out in order to establish a series connection between the unit cells isolated by P1. Boron-doped ZnO, which is a TCO, was deposited on the buffer layer as a window layer via low-pressure chemical vapour deposition. As the final step, scribing for the third pattern (P3) was carried out and the TCO/Buffer/CIGSSe layers were cut.

### Measurements

The microstructures and morphologies of the thin films were observed by field emission scanning electron microscopy (FE-SEM, Hitachi SU8010). The composition and thickness of the thin films were monitored by In-line XRF at the 5G-scale. The depth profile of the thin film was obtained by SIMS. To evaluate the characteristics of the CIGSSe solar cells with the two buffer layers, their illuminated I–V characteristics were measured using an AM 1.5 G solar simulator at 25 °C. The shunt resistance of the solar cells was defined by the Sunshade method^7^. Shunt resistances of coupon solar cells were measured by fitting the dark I–V curve using a one-diode model. The EL of the solar cells was measured using a Si-based charge-coupled device. The DLIT of the solar cells was measured using a mercury cadmium telluride sensor (ImageIR Series, 640 × 512 pixel, InfraTec) and a cooling stage, which maintained the temperature of the solar cells at 25 °C. The lock-in parameters such as the frequency (100 Hz to 1 KHz), duty (50%), and integration time (5 min) were adjusted to obtain the high-resolution image of the defects.

## Supplementary information

Supplementary Information

## Data Availability

The datasets supporting the conclusions of this article are included within the article and the supplementary data file.
