# Supplementary material for: Impact of Buffer Layer Process and Na on Shunt Paths of Monolithic Series-connected CIGSSe Thin Film Solar Cells
Source: Sci Rep. 2019 Mar 6;9:3666. doi: 10.1038/s41598-019-38945-5 (PMC6403329; doi:10.1038/s41598-019-38945-5)
Supplement: Supplementary file 1 — Supplementary Information [file 41598_2019_38945_MOESM1_ESM.docx]

Impact of Buffer Layer Process and Na on Shunt Paths of Monolithic Series-connected CIGSSe Thin Film Solar Cells

Chan Bin Mo^1, 2^, Se Jin Park^1^, Soohyun Bae^1^, Mi-hwa Lim^3^, Junggyu Nam^3^, Dongseop Kim^3^, JungYup Yang^4^, Dongchul Suh^5^, Byoung Koun Min^6^, Donghwan Kim^1^, Yoonmook Kang^6,*^ Young-Su Kim^3,*^ and Hae-seok Lee^6,*^

^1^ Department of Materials Science and Engineering, Korea University, 145 Anam-ro, Seongbuk-gu, Seoul, 136-701, Republic of Korea

^2^ Gangwon Regional Division, Korea Institute of Industrial Technology, Gangneung-Si, Gangwon-Do 210-340, Republic of Korea

^3^ Photovoltaic Development Team, Samsung SDI Co., Ltd., Cheonan-Si, 331-300, Republic of Korea

^4^Department of Physics, Kunsan National University, 54150, Republic of Korea

^5^Department of Chemical Engineering, Hoseo University, Asan, Republic of Korea

^6^ KU-KIST Green School, Graduate School of Energy and Environment, Korea University, 145 Anam-ro, Seongbuk-gu, Seoul, 136-701, Republic of Korea

* ddang@korea.ac.kr, Tel.: (82)-2-3290-5913, Fax: (82)-2-928-3584


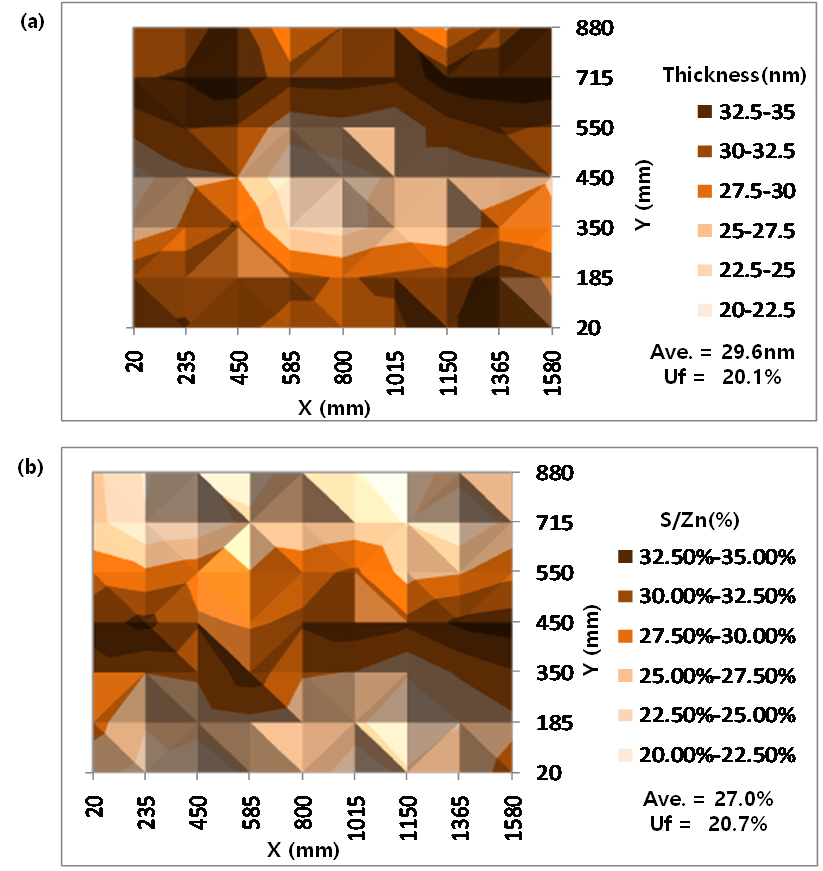


**Supplementary Fig. S1.** Thickness and composition of ALD buffer measured by XRF (a) Thickness and (b) composition (S/Zn) of ALD buffer layer calculated where ratio of Zn/(S+O) is 1.


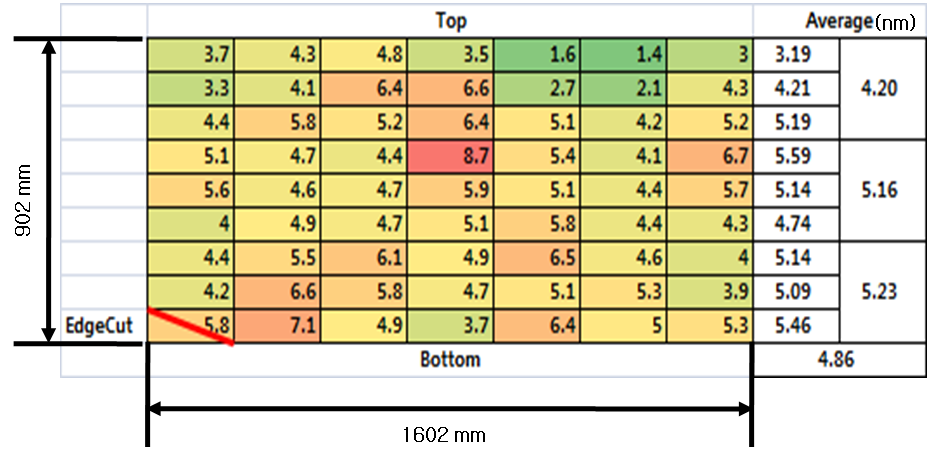


**Supplementary Fig. S2**. Thickness of CBD buffer layer measured by XRF.


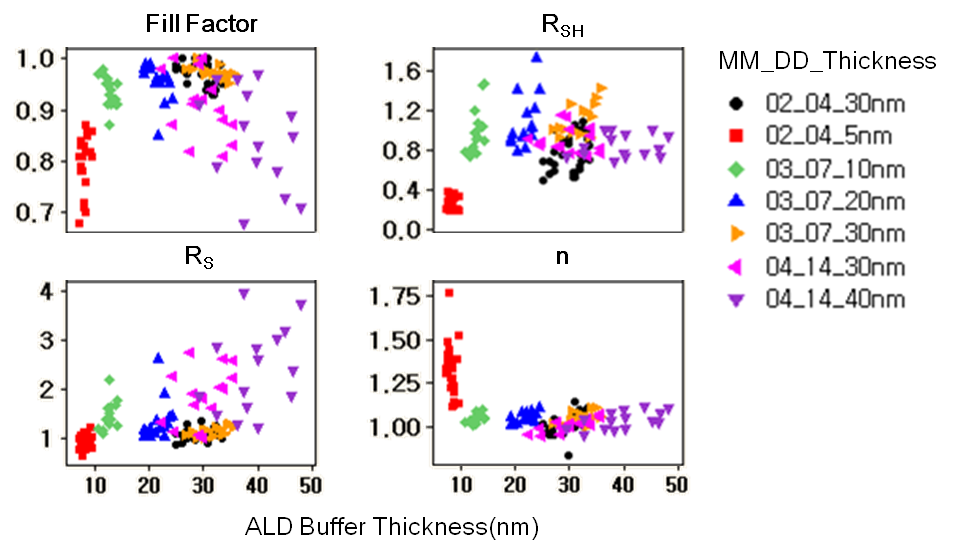


**Supplementary Fig. S3**. Nomarlized fill factor, shunt resistance(R_SH_), series resistance(R_S_) and diode ideality factor(n) with respect to ALD buffer thicknesses (5nm, 10nm, 20nm, 30nm, 40nm)

**Supplementary Table S1**. Fill factor loss calculated from shunt resistance, series resistance and diode ideality factor when V_OC_ is 0.66V, J_SC_=33mA/cm^2^, diode ideality factor=1.5 which are similar to values from I-V measurements of CIGSSe solar cells with the CBD and ALD buffers.

| **R_SH_ (Ωcm^2^)** | **10,000** | **5,000** | **2,000** | **1,000** | **900** | **800** | **700** | **600** | **500** |
| --- | --- | --- | --- | --- | --- | --- | --- | --- | --- |
| **FF_Ideal_ (%)** | **83.89** | **83.89** | **83.89** | **83.89** | **83.89** | **83.89** | **83.89** | **83.89** | **83.89** |
| **FF_0_ (%)** | **78.46** | **78.46** | **78.46** | **78.46** | **78.46** | **78.46** | **78.46** | **78.46** | **78.46** |
| **FF_SH_ (%)** | **78.34** | **78.21** | **77.82** | **77.18** | **77.04** | **76.86** | **76.63** | **76.33** | **75.90** |
| **FF_0_-FF_SH_ (%)** | **0.13** | **0.26** | **0.64** | **1.28** | **1.42** | **1.60** | **1.83** | **2.14** | **2.56** |

**Supplementary Table S2**. Shunt resistances measured and calculated for four different coupon samples of CIGSSe solar cells with different buffer layers where R_SH_meas_ is initial shunt resistance measured as they are prepared, R_SH,P3_meas_ is shunt resistance measured after removal of TCO on P3 region, R_SH_(P3) is shunt resistance contribution of P3 region calculated by 1/{1/(R_SH_meas_·A)-1/(R_SH, P3_meas_·A')}·A, R_SH,0_meas_ is shunt resistance measured after removal of TCO on P1 and P3 region and R_SH_(P1) is shunt resistance contribution of P1 region calculated by 1/{1/(R_SH,P3_meas_·A')-1/(R_SH,0_meas_·A_0_)}·A and R_SH_(P1, P3) is calculated by 1/(1/R_SH_(P1) + 1/R_SH_(P3)). A, A' and A_0_ are areas of coupons.

| Buffer. | Coupon  No. | R_SH_meas_  (Ωcm^2^, A=0.44cm^2^) | R_SH,P3_meas_  (Ωcm^2^, A'=0.39cm^2^) | R_SH,0_meas_  (Ωcm^2^, A_0_=0.34cm^2^) | R_SH_(P1)  (Ωcm^2^) | R_SH_(P3)  (Ωcm^2^) | R_SH_(P1, P3)  (Ωcm^2^) |
| --- | --- | --- | --- | --- | --- | --- | --- |
| CBD | #1 | 1194 | 3785 | 12581 | 5788 | 1657 | 1288 |
|  | #2 | 1141 | 4520 | 24616 | 6072 | 1470 | 1183 |
|  | #3 | 1157 | 4632 | 23313 | 6321 | 1486 | 1203 |
|  | #4 | 1283 | 4967 | 10175 | 9754 | 1663 | 1421 |
| ALD | #1 | 847 | 5717 | 41349 | 7334 | 975 | 861 |
|  | #2 | 790 | 3012 | 172858 | 3451 | 1029 | 793 |
|  | #3 | 743 | 2062 | 75527 | 2383 | 1092 | 749 |
|  | #4 | 657 | 2106 | 92851 | 2424 | 909 | 661 |
